# Use of Hypnosis in Paediatric Gastrointestinal Endoscopy: A Pilot Study

**DOI:** 10.3389/fped.2021.719626

**Published:** 2021-09-22

**Authors:** Léa Chantal Tran, Stéphanie Coopman, Céline Rivallain, Madeleine Aumar, Dominique Guimber, Audrey Nicolas, Valérie Darras, Dominique Turck, Frédéric Gottrand, Delphine Ley

**Affiliations:** ^1^Univ. Lille, Inserm, CHU Lille, U1286 - INFINITE - Institute for Translational Research in Inflammation, Lille, France; ^2^Division of Gastroenterology, Hepatology and Nutrition, Department of Paediatrics, Jeanne de Flandre Children's Hospital, CHU Lille and Univ. Lille, Lille, France

**Keywords:** hypnosis, endoscopy, children, EMONO, midazolam

## Abstract

**Objectives:** Experience of hypnosis in gastrointestinal (GI) endoscopy is scarce in children. Our aims were to assess the rate of successful GI endoscopy performed using hypnosis alone or in combination with midazolam, with or without additional equimolar mixture of oxygen and nitrous oxide (EMONO), and to identify predictive factors of successful endoscopy in children.

**Methods:** This prospective single-centre study included children older than 6 years requiring a diagnostic esophagogastroduodenoscopy (EGD) or rectosigmoidoscopy. Ericksonian hypnosis was performed alone or in combination with midazolam, with or without additional EMONO. Successful endoscopy was defined by a complete and well-tolerated procedure. Levels of satisfaction of the endoscopist, nurse, and patient were assessed.

**Results:** One hundred forty children [70 boys, median age: 12 years (Q1–Q3: 9–14)] were included over a 14-month period. They underwent EGD in 51.4% (*n* = 72) and rectosigmoidoscopy in 48.6% (*n* = 68) of cases. EMONO and midazolam were combined with hypnosis in 136 cases (97.1%). Successful endoscopy rate reached 82.9%. The procedure was interrupted due to poor tolerance and was rescheduled under general anaesthesia in 11 patients (7.9%). Predictive factors for successful endoscopy were older age (13 vs. 8 years, OR: 1.34, CI 95% [1.10–1.62], *p* = 0.003) and type of endoscopy (EGD vs. rectosigmoidoscopy, OR: 16.34 [2.14–124.68], *p* = 0.007). A good cooperation of the patient was reported by the endoscopist and the nurse in 88.4 and 86.9% of cases, respectively. Ninety-two per cent of patients mentioned that the procedure went well.

**Conclusions:** Our study suggests that hypnosis combined with EMONO and/or midazolam is of additional value to perform diagnostic EGD or rectosigmoidoscopy in children older than 6 years without systematic need for general anaesthesia.

## Introduction

Pain triggered by gastrointestinal (GI) endoscopy is, such as any pain, multidimensional and encompasses sensorial and emotional fields. Anxiety is an emotion close to painful experience, as it can increase the perception of pain. This situation commonly observed among children has been also seen in adult studies where scores of anxiety and pain often have a positive correlation (1, 2).

Hypnosis deals with a natural state of modified conscience involving focused attention and reduced peripheral awareness, allowing an enhanced ability to respond to suggestions (3). In clinical practice, hypnosis guided by a trained practitioner aims to change pain and anxiety perception of the patient using his/her mental resources, in order to improve comfort. Even if the practice of hypnosis in daily care is still rare, it has been considered as a valuable alternative in various clinical situations (4). Many studies have shown its efficacy in the management of pain but also anxiety among children (5, 6). In 2005, Calipel et al. demonstrated the efficacy of hypnosis on anxiety as premedication before surgery, comparing hypnosis and oral midazolam in a randomised controlled trial (RCT) involving 50 children from 2 to 11 years of age (5). Children who were under hypnosis were significantly less anxious than those who received midazolam and had significantly less behaviour disorders on days 1 and 7 after surgery. In 2009, another RCT showed the benefits of hypnosis combined with a local anaesthetic (EMLA^®^) compared with distraction combined with the same anaesthetic on venepuncture-induced pain in 45 children affected with cancer (6). Patients from the former group displayed less anticipatory anxiety and less behavioural distress during the intervention. A Cochrane meta-analysis published in 2018 by Birnie et al. reviewed the efficacy of distraction and hypnosis to reduce needle-related pain and distress among children and adolescents (7). Among the eight included RCT dealing with hypnosis, five studies including 176 participants showed a statistically significant effect of hypnosis on self-reported pain. Because of pain and anxiety, GI endoscopy under conscious sedation is usually not well-tolerated. While complications during GI endoscopies under general anaesthesia are generally scarce, especially in children, they are known to occur more frequently in the presence of patient risk factors, such as anxiety (8, 9). In adults, several studies pointed out the efficiency of hypnosis in the reduction of pain and anxiety during invasive procedures including GI endoscopy (10–12). The effectiveness of hypnosis compared with intravenous sedation in esophagogastroduodenoscopy (EGD) is still a matter of debate in adults (13, 14). To our knowledge, no paediatric study reported the use of hypnosis during GI endoscopy.

We conducted a prospective pilot study with the primary objective to assess the rate of successful GI endoscopy performed using hypnosis alone or in combination with midazolam, with or without equimolar mixture of oxygen and nitrous oxide (EMONO) in children. The secondary objectives were to identify predictive factors of successful GI endoscopy and to evaluate the level of satisfaction of children, nurses, and endoscopists with regard to the procedure.

## Methods

### Patients

We conducted a prospective, monocentric pilot study over a 14-month period. All patients aged between 6 and 18 years for whom a GI endoscopy was scheduled at the Lille University Jeanne de Flandre Children's Hospital were considered for inclusion. For patients who underwent several GI endoscopies during the study period, only the first GI endoscopy procedure performed was selected for analysis. Exclusion criteria included age below 6 years, deafness without hearing aids, and/or cognitive disorders, corresponding to situations when hypnosis could not be fully understood. Cases of emergency procedure and cases when patients and/or their parent/guardian were not willing to participate were also excluded.

### Endoscopic Procedure Under Hypnosis

Endoscopic procedures included diagnostic EGD and rectosigmoidoscopy. EGD associated with ileocolonoscopy and interventional EGD were systematically performed under general anaesthesia for patient's comfort and safety and therefore were not considered in the present study. GI endoscopy procedures were performed by seven experienced senior paediatric gastroenterologists, with a mean of 20 procedures per endoscopist during the study. Flexible video-endoscopes from PENTAX^®^ or OLYMPUS^®^ were used according to the patient's weight. The three nurses from the paediatric endoscopy unit were qualified to perform hypnosis (national certificate in hypnoanalgesia and distraction). Hypnosis was administered before the procedure by one nurse according to an Ericksonian approach. The Ericksonian approach relies on the child's imagination to allow him/her to escape and change the perception of the procedure. The Ericksonian approach uses verbal and non-verbal indirect suggestions, adapted on the child's reaction, to induce behavioural change (15). A hypnosis session started with the induction of the hypnotic condition by capturing the patient's attention and saturating his/her mind with sensory suggestions. The success of the hypnotic induction was assessed by the nurse who evaluated the state of deepening in which the patient kept the ability to answer to simple orders. Then, the patient underwent a dissociation of his/her real perception, before returning to ordinary sensoriality at the end of the procedure. The patient could choose to have GI endoscopy either with hypnosis alone (a) or with sublingual midazolam (b). Once installed on the examination table, he could choose to have additionally EMONO (c) or not (d). The dosage of midazolam depended on the patient's body weight, with a maximum of 10 mg (0.35 mg/kg for body weights <30 kg and 0.15 mg/kg for body weights >30 kg).

Successful endoscopy was defined as a complete procedure (i.e., not stopped before the end and when all planned biopsies were done), which was judged well tolerated by the patient. The procedure was assessed as complete or not by the endoscopist, and its tolerance was evaluated by the patient using one closed question (“do you think this procedure went well?”).

### Data Collection

The following data were prospectively collected using a standardised questionnaire specifically designed for the study: age; gender; past history of GI endoscopy; indication for the present GI endoscopy; type of procedure (EGD or rectosigmoidoscopy); presence of at least one parent during the exam; time spent in the waiting room; time between arrival in the endoscopy room and beginning of hypnosis; level of patient's anxiety before the procedure (“not at all”, “a little”, “a lot”, and “very much”); time spent in the endoscopy room; the use of midazolam and/or EMONO; duration of endoscopy and hypnosis; proportion of procedures requiring conversion to general anaesthesia; level of satisfaction of the patient, the nurse, and the endoscopist about the procedure (“good” or “bad”); patient's cooperation and pain caused by the endoscopy according to the patient using a Visual Analogue Scale; procedure performance; and proportion of biopsies performed when compared with the number of initially planned biopsies.

### Statistical Analysis

Quantitative variables were described by mean values and standard deviations or median and interquartile range. Gaussian distribution of continuous variables was tested by the Shapiro–Wilk test. Qualitative variables were described by frequencies and percentages. Quantitative variables were compared by Student's *t*-test, and Wilcoxon non-parametric test was used in case of non-normality of the data. Categorical variables were compared by chi-square test or Fisher's exact test if *n* < 5. Factors associated with successful endoscopy in univariate analysis with a *p*-value < 0.1 were included in a multivariate model. SAS software version 9.4^®^ (Cary, NC, USA) was used for the analyses. A *p*-value < 0.05 was considered significant.

### Ethics

The research work was conducted in accordance with protocols, good clinical practice, and the relevant laws and regulations in France and did not need institutional review board (IRB) approval. Several days prior to the procedure, a preliminary information was given by phone to the family. At the day of the exam, an information letter and a written consent form were given to the patient, and his/her parents and/or guardian. In case of opposition, data were not collected or were immediately removed from the database. The study had an agreement from the Commission Nationale de l'Informatique et des Libertés (CNIL-French Data Protection Authority).

## Results

### Study Population

**Figure 1:**
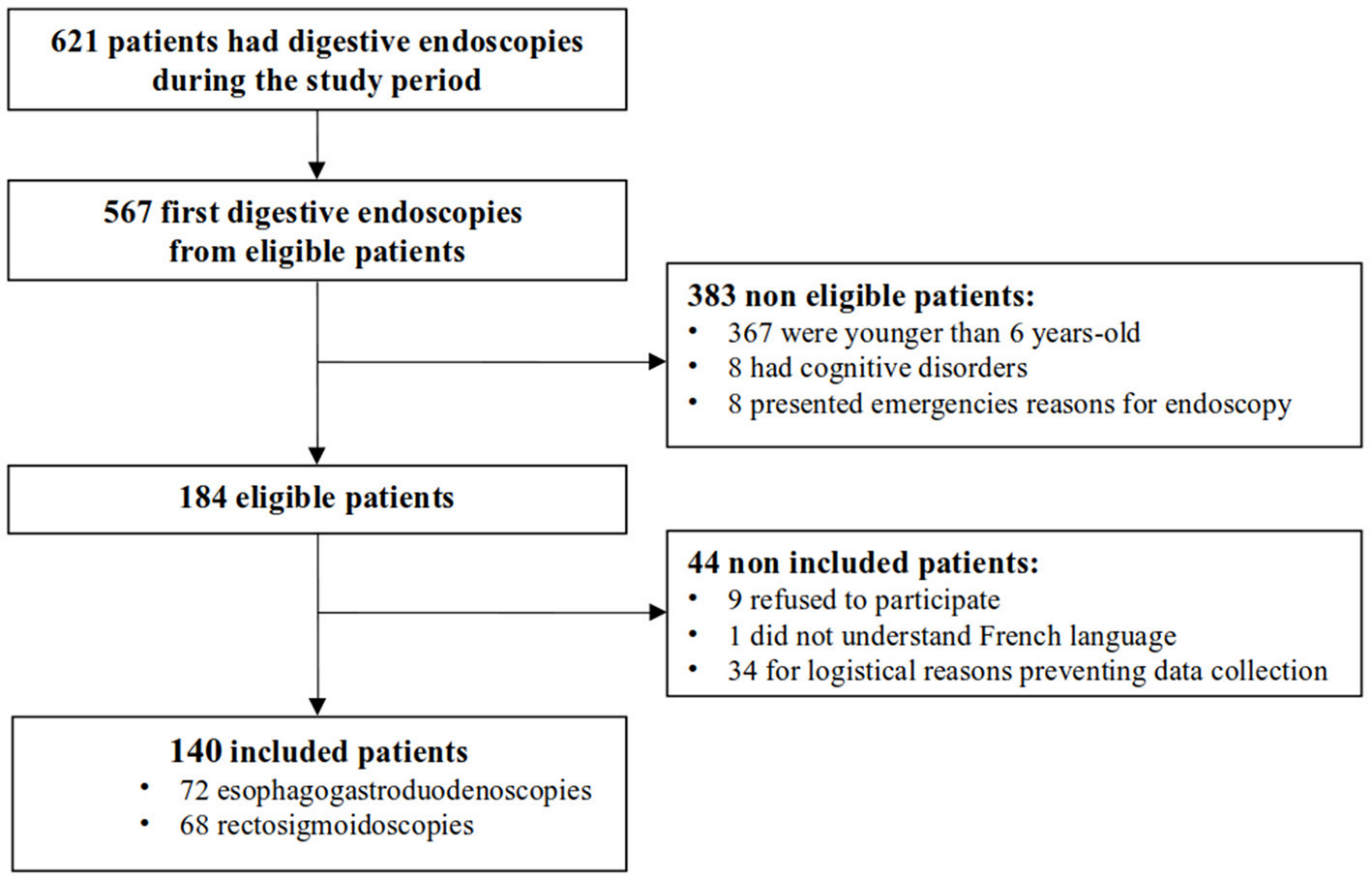
Study flow-chart.

One hundred eighty-four patients older than 6 years requiring a diagnostic EGD and rectosigmoidoscopy were considered for inclusion, which corresponded to 29.6% of the 621 patients who underwent GI endoscopy during the study period. Of these, 44 patients met exclusion criteria: nine refused to participate, one did not understand French, and data of 34 could not be collected. A total of 140 patients were included (Figure 1). One patient had both EGD and rectosigmoidoscopy during the same procedure. Compared with non-included patients, EGD was more frequently performed than rectosigmoidoscopy in included patients (51.4 vs. 22.7%, *p* < 0.001), mostly following an indication of abdominal pain (35.0 vs. 13.6%, *p* = 0.012) or gastroesophageal reflux/vomiting (25.7 vs. 6.8%, *p* = 0.0059). Conversely, rectosigmoidoscopy was less performed among included patients who were suspected of inflammatory bowel disease or had chronic diarrhoea (29.1 vs. 56.8%, *p* < 0.001) (Table 1).

**Table 1:** Demographic characteristics and indications for gastrointestinal endoscopy in included and excluded patients.

	**Eligible and included patients** **(*n* = 140)**	**Eligible and excluded patients** **(*n* = 44)**	***p*-value**
Male, *n* (%)	70 (50.0)	18 (40.9)	0.29
Median age (Q1–Q3)	12 (9.0–14.0)	12 (8.0–14.0)	0.36
Used sedation for past^*^ GI endoscopy, *n* (%)	53 (37.9)	17 (38.6)	0.93
- General anaesthesia	32 (23.0)	12 (27.3)	0.57
- Hypnoanalgesia	21 (15.3)	5 (11.6)	0.55
- Sedation with EMONO and/or midazolam	30 (22.1)	8 (19.0)	0.68
Esophagogastroduodenoscopy, *n* (%)	72 (51.4)	10 (22.7)	** <0.001**
- Abdominal pain	49 (35.0)	6 (13.6)	**0.012**
- Gastroesophageal reflux/vomiting	36 (25.7)	3 (6.8)	**0.0059**
- Feeding difficulties	15 (10.7)	2 (4.5)	0.37
- Follow-up of known lesions	5 (3.6)	0 (0)	0.34
- Weight loss/failure to thrive	5 (3.6)	2 (4.5)	0.67
- Digestive haemorrhage	2 (1.4)	2 (4.5)	0.24
- Celiac disease	2 (1.4)	1 (2.3)	0.56
- Inflammatory bowel disease/chronic diarrhoea	1 (0.7)	0 (0)	1
- Other indication	0 (0)	1 (2.3)	0.24
Rectosigmoidoscopy, *n* (%)	69 (49.3)^**^	34 (77.3)	**0.002**
- Inflammatory bowel disease/chronic diarrhoea	38 (29.1)	25 (56.8)	** <0.001**
- Digestive haemorrhage	18 (12.9)	7 (15.9)	0.79
- Follow-up of known lesions	9 (6.4)	1 (2.3)	0.45
- Abdominal pain	5 (3.6)	2 (4.5)	0.67
- Other indication	5 (3.6)	0 (0)	0.34

*GI, gastrointestinal; EMONO, equimolar mixture of oxygen and nitrous oxide*.

* *Previous GI endoscopy before the study period*.

** *One patient had both esophagogastroduodenoscopy and rectosigmoidoscopy during the same procedure*.

*The bold values correspond to significant p-values (p < 0.05)*.

### Rate of Successful Endoscopy

Hypnosis was combined with sedation in 136 cases (97.1%) (Table 2). Mean time (±SD) between entering the endoscopy room and beginning of hypnosis was 15.6 (±8.9) min. GI endoscopy started after a mean time of 4.7 (±2.9) min after the hypnotic induction. Mean duration of GI endoscopy was 6.2 (±3.2) min, while the mean hypnosis duration was 12.7 (±5.4) min. Mean time between the end of the procedure and release from the endoscopy room was 8.0 (±4.0) min. Biopsies were planned by the endoscopist before the procedure in 86.9% of cases, and all planned biopsies were harvested. The mean (±SD) number of planned biopsies per patient was 4.1 (±2.6); and the mean number of biopsies harvested was 4.2 (±3.0). GI endoscopy was successful in 116 patients (82.9%) and failed in 24 patients (17.1%). The four procedures performed under hypnosis alone were all successful rectosigmoidoscopies, performed on two girls aged 6 years and two boys aged 8 and 12 years. The rate of successful GI endoscopy was 93.8% when hypnosis was combined with EMONO (*n* = 60/64) and 71.8% when hypnosis was combined with EMONO and midazolam (*n* = 51/71). Among the failed procedures, three were associated with a poor tolerance of the patient, and 13 were stopped because of a poor tolerance of the procedure according to the endoscopist, and/or all biopsies could not be obtained. The endoscopy procedure had to be rescheduled on general anaesthesia in 11 cases. Rates of successful GI endoscopy were similar between two nurses (91.1 and 87.7%) and were lower for the third nurse (72.9%), who practiced more often EGD (59.3%) than the two first nurses (53.3 and 33.3%). The range of success rate of the seven endoscopists comprised between 62.5 and 100%.

**Table 2:** Sedation used in combination with hypnosis during gastrointestinal endoscopy in included patients.

	**All GI endoscopies** **(*n* = 140)**	**EGD** **(*n* = 72)**	**Rectosigmoidoscopies** **(*n* = 68)**
**Hypnosis combined**			
**with sedation** ***n*** **(%)**			
- EMONO	68 (48.6)	1 (1.4)	67 (98.5)
- Midazolam	1 (0.7)	0 (0.0)	1 (1.5)
- EMONO and midazolam	71 (50.7)	71 (50.7)	0 (0.0)

*GI, gastrointestinal; EGD, esophagogastroduodenoscopy; EMONO, equimolar mixture of oxygen and nitrous oxide*.

### Level of Satisfaction With Endoscopy Under Hypnosis

Patients showed a good cooperation according to the endoscopist and the nurse in 88.4 and 86.9% of cases, respectively. Ninety-two per cent of patients mentioned that the procedure went well. When considering the possibility of repeating the procedure under hypnosis, scores were consistent between the endoscopist, the nurse, and the child (81.9, 83.1, and 81.2% of positive answers, respectively), with 80.7% (*n* = 113) of doctors and nurses and 81.4% (*n* = 112) of patients who would do it again. Among individuals who would repeat the intervention, 96.5% (*n* = 109/113) of doctors and nurses and 88.8% (*n* = 103/112) of patients experienced a successful endoscopy.

Before the procedure, 68.3% of patients described anxiety, while nurses considered 76.2% of patients as anxious. Assessment of anxiety intensity was significantly different between patients and nurses (*p* = 0.003): 38.1% of patients perceived anxiety as mild (vs. 27% of nurses), 15.9% as moderate (vs. 20.6% of nurses), and 14.3% as severe (vs. 28.6% of nurses). Patients declared feeling pain during the exam in 70.1% of the cases, with median evaluated pain at 2.5 (min–max: 0.0–5.0). The evaluated pain was lower in the successful group than in the failure group (2.0 (0.0–4.0) vs. 5.0 (3.0–7.5), *p* < 0.001, respectively). Median pain was evaluated at 3.0 (0.0–5.5) for EGD and 2.0 (0.0–4.0) for rectosigmoidoscopy.

### Predictive Factors of Successful Gastrointestinal Endoscopy

Children in the successful group were older than those in the failure group (median age of 13 vs. 8 years, *p* < 0.001) in the univariate analysis (Table 3). There were more cases of failure with EGD compared with rectosigmoidoscopy (83.3 vs. 16.7%, *p* < 0.001). Median time between entrance in the endoscopy room and beginning of the hypnosis was significantly lower in the successful group than in the failure group (14.8 vs. 19.4 min, *p* = 0.03).

**Table 3:** Predictive factors of successful gastrointestinal endoscopy.

	**Success group** **(*n* = 116)**	**Failure group** **(*n* = 24)**	***p*-value**	**Adjusted OR**	**95% CI**	***p*-value**
Male, *n* (%)	54 (46.6)	16 (66.7)	0.07	–	–	–
Median age (Q1–Q3)	13.0 (10.0–14.5)	8.0 (7.0–11.5)	** <0.001**	1.34	[1.10–1.62]	**0.003**
History of digestive endoscopy, *n* (%)	46 (39.7)	7 (29.2)	0.33	–	–	–
Presence of anxiety before the exam, *n* (%)	76 (69.1)	19 (79.2)	0.32	–	–	–
Intensity of anxiety before the exam, *n* (%)	–	–	0.09			
Absence	34 (30.9)	5 (20.8)	–			
Mild	50 (45.5)	12 (50.0)	–			
Moderate	12 (10.9)	0 (0.0)	–			
Severe	14 (12.7)	7 (29.2)	–			
Presence of parents during the procedure, *n* (%)	73 (64.6)	10 (43.5)	0.06	–	–	–
Esophagogastroduodenoscopy, *n* (%)	52 (44.8)	20 (83.3)	** <0.001**	16.34	[2.14–124.68]	**0.007**
Required biopsies, *n* (%)	99 (86.1)	20 (90.9)	0.74	–	–	–
Time spent in the waiting room (mean in min ± SD)	36.0 ± 24.8	49.6 ± 43.0	0.27	–	–	–
Time between the entrance in the endoscopy room and the beginning of hypnosis	14.8 ± 9.1	19.4 ± 7.4	**0.03**	1.06	[0.96–1.17]	0.27
(mean in min ± SD)						

*OR, odds ratio; 95% CI, 95% confidence interval*.

*The bold values correspond to significant p-values (p < 0.05)*.

In multivariate analysis, success of the endoscopy was associated with age of the children (13 vs. 8 years, OR: 1.34, CI 95% [1.10–1.62], *p* = 0.003) and type of procedure (rectosigmoidoscopy vs. EGD, OR: 16.34 [2.14–124.68], *p* = 0.007). An additional year of age was associated with 1.33 times more likelihood to have a successful procedure. After adjustment of age and time between entrance in the endoscopy room and beginning of the hypnosis, there were 16 times more cases of failure in EGD than in rectosigmoidoscopy.

Factors associated with successful EGD in univariate analysis were older age (12 vs. 9 years, *p* = 0.001) and presence of parents during the procedure (60.8 vs. 31.6%, *p* = 0.029), whereas male gender was significantly associated with cases of failure (40.4 vs. 70.0%, *p* = 0.024). In multivariate analysis, only patient's age and presence of parents were significantly associated with the success of EGD. There were no differences in success or failure of hypnosis if parents where present (*n* = 83) or not (*n* = 53). When considering only successful endoscopies, no difference was found (*n* = 73 and *n* = 43).

## Discussion

This is the first prospective study reporting the use of hypnosis during GI paediatric endoscopy in a large number of patients. Overall, we observed a high success rate of GI endoscopy under hypnosis combined with sedation induced by EMONO and/or midazolam. Four rectosigmoidoscopies were performed with hypnosis alone and were all successful. Older age and rectosigmoidoscopy were significant predictive factors associated with success of endoscopy.

Previous studies reported the use of hypnosis without sedation during GI endoscopy in adult patients. Cadranel et al. used hypnotic relaxation to perform colonoscopy in 24 patients with a mean age of 43 years (16). Hypnosis resulted in moderate or deep sedation in half of them. Pain was lower when hypnosis was successful. In addition, completeness of colonoscopy was observed in all patients in the successful group as compared with only half of them in the failure group. Dominguez-Ortega et al. observed an efficacy of hypnosis used alone in EGD (*n* = 6) and colonoscopy (*n* = 22), with a good tolerance reported by the patient in 85% of cases (17). Elkins et al. studied the effect of hypnosis in the management of pain and anxiety during colonoscopy performed for colorectal cancer screening. Patients having a hypnotic induction had lower anxiety before the procedure, reduced recovery time after the procedure, lower vasovagal events, and a high level of satisfaction of the endoscopic procedure compared with the patients without hypnosis. Successful hypnosis was associated with less intense pain as compared with failed hypnosis (18). In a preliminary report on patients who underwent colonoscopy under hypnosis (*n* = 38) or midazolam (*n* = 29), Bersani et al. showed less pain (Visual Analogue Scale 2.97 vs. 5.48, *p* < 0.05) and higher satisfaction (63 vs. 24%, *p* < 0.05) in the hypnosis group compared with the midazolam group (19). Other authors pointed out the efficiency of hypnosis as part of a psychological preparation to GI endoscopies, in order to reduce pre-operative anxiety (20, 21).

In the present study, older age was statistically associated with successful endoscopy. This predictive factor could be expected since older children better understand and apprehend the course of the procedure. The median age in the successful group was 13 years (vs. 8 years). According to Wood and Bioy, age is a major criterion to consider in hypnosis since children are more likely to be receptive to hypnosis between 7 and 14 years of age (22). For Olness and Kohen, the ability to be hypnotised is limited before the age of 3 years, reaches a peak between 7 and 14 years, and decreases during adolescence, followed by stabilisation and a final decrease at maturity (23). Healthcare situations may be stressful for younger children, yet fear increases the perception of pain. In multivariate analysis, successful endoscopy was strongly influenced by the type of GI endoscopy since there were more cases of failure in EGD compared with rectosigmoidoscopy. This difference is very likely associated with a lower tolerance and higher level of stress with regard to EGD by patients in comparison with rectosigmoidoscopy, independently of hypnosis efficacy (24). Our results revealed more cases of successful GI endoscopies when patients used hypnosis combined with EMONO than patients using hypnosis combined with EMONO and midazolam. The higher success rate in the first group can be explained by a higher number of rectosigmoidoscopies (98.3%) when all procedures from the second group were EGD, but also more parental presence (66.7 vs. 60.8%). Median time between entrance in the endoscopy room and beginning of the hypnosis was significantly shorter in the success group. Older age and more frequent parental presence may have influenced this difference. We assumed that older patients may have required less time to understand the procedure and have been more easily reassured. The presence of parents during EGD was associated with successful endoscopy. In addition to a preparation session when clear information is given to parents and their child, parental presence is known to participate in the relief from child pre-operative stress (25). In a study including 42 adults who underwent EGD, alone or accompanied, it was shown that patients with a guide tended to have lower anxiety than those without, with a higher benefit when the patients had a higher level of anxiety before the procedure (26). In an RCT on 130 children who underwent painful procedures, significant decreases in scores on pain experience and stress were observed in the parental presence group compared with the group of children using a kaleidoscope toy or the control group (without parents) (27). One could expect a lower efficiency of hypnosis during GI endoscopy requiring multiple digestive biopsies, or in very anxious children. However, in our study, the child's pre-existent anxiety, history of previous endoscopy, or requirement of biopsies did not influence the rate of successful endoscopy.

With a monocentric recruitment, this study offered the advantage of displaying a homogeneous patient care. However, the study lacked statistical power, particularly regarding the assessment of predictive factors of successful endoscopy due to the small number of patients in the failure group. For this reason, the roles of the endoscopist and the nurse who performed hypnosis have not been evaluated as a prognostic factor of successful endoscopy. However, the range of success rate of the seven endoscopists did not vary significantly. The low failure rate may be explained by the evaluation of success with one closed question, preventing a more precise graduated answer. The study design with a planned protocol could also have implied the caregivers to explain the procedures to the patient and his/her family more carefully than usual. Plus, when considering the median age, our study population could have been particularly sensitive to hypnosis success, as detailed above (22, 23). The definition of success was arguable as we randomly chose to consider the endoscopist's and the patient's point of view, even if the judgment criteria were defined to be as objective as possible. The evaluation of both anxiety and satisfaction could have been standardised using validated and blinded questionnaires. We did not assess the long-term effects of hypnosis since we did not expect any long-term adverse event after discharge from the hospital, although this is a limitation of our study. To our knowledge, no patients reported long-term events after the study. We regret that some patients could not be included because of logistical reasons, such as the insufficient number of trained caregivers to perform hypnosis. We did not assess patient's hypnotisability or the depth of hypnotic state, which could have been interesting to compare between different age groups. Finally, the study was designed to be observational, allowing assumptions about the benefit of hypnosis during paediatric GI endoscopy only and hindering the establishment of causality. We did not choose to compare GI endoscopy with and without hypnosis, but we compared different modalities of sedation combined with hypnosis. This question could be clarified in further studies using a higher number of randomised patients, allowing comparisons with a control group. However, since the success rate using combination of hypnosis and sedation is very high in our pilot study, we do believe this technique is of interest for clinical practice.

Currently, the shortage of anaesthesiologists urges the development of alternatives to general anaesthesia for effective sedation in children. The choice of sedative drugs is large (e.g., propofol, ketamine, and midazolam), but none of them possesses all the ideal properties: quick efficacy, predictable dose-dependent effect, large therapeutic window, anxiolytic effect with anterograde amnesia during the exam, quick half-life, and minimal side effects (28). Moreover, the use of sedative drugs by doctors other than anaesthetists is not allowed in many countries. Sedation represents a continuum going from mild to deep sedation; therefore, there is always a risk of involuntarily move from a mild to deep level of sedation with loss of airway protection reflexes, respiratory depression, and haemodynamic instability. Sedation procedure must offer an effective and safe alternative to general anaesthesia. Hence, hypnosis combined with conscious sedation, such as sub-lingual midazolam and EMONO, is an interesting sedative choice for GI endoscopy.

Several conditions are required to apply hypnosis in paediatric GI endoscopy in clinical practice. Members from the medical team have to be trained and habilitated to practice hypnosis. The endoscopy room needs to be adapted for hypnosis, including the reduction of external stimuli (light and noise) to create a quite atmosphere. The targeted population receiving hypnosis has to be selected: diagnostic EGD or rectosigmoidoscopy and age older than 6 years. When children arrived in the endoscopy room, a clear information has to be delivered to the children and their parents/guardians, to decide an individualised choice of hypnosis alone or associated with conscious sedation. The GI endoscopy procedure can be performed only when the distraction of the children is obtained. Child's satisfaction must be evaluated after the procedure.

This prospective pilot study suggests that hypnosis combined with midazolam and/or EMONO is an effective technique and may be of additional value to increase the success and tolerance of diagnostic GI endoscopy in children older than 6 years. The use of hypnosis represents a complementary tool for patient's sedation with the ambition to transform a care experience into a moment of pleasant escape for the child. By changing communication with the child and renewing the caregivers' routine organisation, hypnosis would thus be integrated into an improved conception of paediatric care.

## Data Availability Statement

The raw data supporting the conclusions of this article will be made available by the authors, without undue reservation.

## Ethics Statement

Ethical review and approval was not required for the study on human participants in accordance with the local legislation and institutional requirements. Written informed consent to participate in this study was provided by the participants' legal guardian/next of kin.

## Author Contributions

SC, CR, VD, and DL conceived and designed the study. LT, SC, CR, and DL did the analysis and interpretation of the data. LT, SC, CR, and DL drafted of the article. SC, MA, AN, DG, DT, FG, and DL brought critical revision of the article for important intellectual content. All authors approved the final version of the article.

## Conflict of Interest

The authors declare that the research was conducted in the absence of any commercial or financial relationships that could be construed as a potential conflict of interest.

## Publisher's Note

All claims expressed in this article are solely those of the authors and do not necessarily represent those of their affiliated organizations, or those of the publisher, the editors and the reviewers. Any product that may be evaluated in this article, or claim that may be made by its manufacturer, is not guaranteed or endorsed by the publisher.
